# Torrefied Agri-Food Residues as a Coal Substitute: How the Supply Chain and the Allocation Rule Determine the Carbon Footprint

**DOI:** 10.3390/ma19214725

**Published:** 2026-11-06

**Authors:** Lukáš Jeníček, Jan Malaťák

**Affiliations:** Faculty of Engineering, Czech University of Life Sciences Prague, Kamýcká 129, 165 00 Prague, Czech Republic; malatak@tf.czu.cz

**Keywords:** carbon footprint, torrefaction, coal substitution, lignite, co-product allocation, system expansion, supply chain, spent coffee grounds, spent tea leaves, nutshells, EU emissions trading system

## Abstract

Agri-food processing leaves large volumes of lignocellulosic residues for which their energy is rarely recovered, while coal-fired heat and power in Europe carries a rising carbon price. This study quantifies the carbon footprint of five food-processing residues—spent coffee grounds (SCGs), spent tea leaves (TEAs) and walnut, pistachio and peanut shells—with spruce wood chips as a non-food benchmark, torrefied at 250–550 °C and burned in place of Czech lignite. Fuel properties were measured for all six materials at five temperatures and their replicate variances propagated. Emissions were quantified for nine process stages—from cultivation of the crop to combustion of the fuel—per tonne of dry residue under a fuel-first allocation that charges the residue with the agricultural chain and credits the food co-product by system expansion; cut-off, mass and economic allocation were compared. All six materials save emissions at their selected temperature, from −557 ± 22 kg CO_2_-eq t^−1^ for SCG to −1214 ± 23 for spruce, avoiding 36–77% of the emissions of the lignite they replace. The ranking is set by the supply chain rather than by the fuel: SCG has the highest calorific value of the set yet saves least, because 89% of its residual burden comes from roasting, brewing and intercontinental freight. The result is conditional: Under mass allocation, four of the five food residues become net emitters, and so do SCG and TEA when collected wet. At 65 EUR tCO_2_^−1^, the avoided emissions are worth 36–79 EUR t^−1^ against a fuel-cost advantage over lignite of 116–200 EUR t^−1^.

## 1. Introduction

The global agri-food system produces vast volumes of lignocellulosic by-products that remain largely unexploited as energy carriers. Coffee, tea and tree nuts are among the most heavily traded commodities, and each leaves a carbon-rich residue: about 0.65 kg of spent grounds per kilogram of green coffee once it has been roasted and brewed [1]; about 0.70 kg of spent leaves per kilogram of tea once it has been infused; and 0.35–0.50 kg of shell per kilogram of in-shell walnuts, pistachios and peanuts [2,3]. These streams have historically been landfilled, composted or anaerobically digested, yet critical reviews of food-waste management increasingly identify thermochemical energy recovery as one of the lowest-impact valorisation routes when the realistic alternative is disposal [4,5]. Their energy content is considerable: the raw spent coffee grounds characterised in our earlier work reached a net calorific value close to 20 MJ kg^−1^, exceeding that of many commercial wood chips [6,7,8].

This substitution matters most for coal, which, in Central Europe above all in the form of lignite, still underpins a substantial share of heat and power and for which its combustion carries a rising cost through the EU Emissions Trading System (EU ETS). A solid biofuel that can be milled and burned in existing coal-fired plants is therefore an attractive drop-in option, and co-firing studies confirm this principle: emissions decline as the co-firing ratio rises, with a torrefaction temperature near 270 °C offering the best balance between energy yield and emission reduction [9,10,11].

Raw biomass, however, is a poor coal substitute: It is bulky, hygroscopic, fibrous and low in energy density. Torrefaction (approximately 200–300 °C) and slow pyrolysis (above 300 °C) drive off moisture and volatiles to yield a hydrophobic, friable, energy-dense solid for which its grindability and combustion behaviour approach those of lignite [12,13]. Our earlier experimental programme mapped this upgrading for all six feedstocks of the present study [6,14,15,16,17,18,19] and showed that the best temperature is set by the trade-off between rising energy density and falling mass yield rather than by the maximum heating value [19,20,21].

Whether an upgraded waste fuel is genuinely climate-beneficial cannot be judged from its calorific value alone. The life-cycle-assessment literature on carbonised biomass has matured rapidly and has shifted from an early emphasis on soil amendment toward carbon footprint and climate change mitigation [22,23]. Several studies report that pyrolysis-based systems can be net carbon-negative [24]: A full-chain assessment of sugarcane bagasse carbonisation reported net savings of about 1.2 t CO_2_-eq per tonne of feedstock [25], and carbonised biomass substituted for coal in steelmaking lowers that sector’s footprint [26]. These conclusions are highly sensitive to methodological choices, particularly the treatment of biogenic carbon and the allocation of burdens between co-products, and one influential analysis shows that the label carbon-negative can hinge entirely on such decisions [27]. Feedstock-specific work echoes this: Cradle-to-gate assessments of spent-coffee-ground pellets and of conventional versus microwave torrefaction report about 0.2 kg CO_2_-eq per kilogram of fuel [28,29], comparative assessments place solid fuel recovery among the more favourable valorisation routes [30], and parallel reviews set out the counter-case for soil application [31].

The gap that remains is one of system boundary rather than feedstock coverage. Existing spent coffee ground assessments stop at the pellet gate or compare valorisation routes, and published co-firing assessments rely on woody residues. Where food residues are assessed, the supply chain is almost invariably modelled as a regional one, even for commodities that reach Europe from Brazil, India or Iran. No cradle-to-grave study yet treats spent coffee grounds, spent tea leaves and nutshells together as dedicated fuel feedstocks; charges them with the logistics of their real place of origin; and benchmarks them against coal under one consistent accounting convention.

The present study addresses this gap from a deliberately conservative fuel-first perspective: The residue is charged with the agricultural chain of its crop, and the food commodity is handled as a co-product that earns a system credit. Relative to the cut-off and economic conventions, this is a demanding test, and all six materials pass it by margins that differ by a factor of 2.2; mass allocation, also reported, is stricter still. Four features distinguish the assessment from its predecessors: The thermochemical data are primary measurements on all six materials; Stage 3 transport is built from origin-specific sea, road and rail routes rather than from an assumed haulage distance; drying energy is computed from the received moisture of each stream; the allocation convention and the scope of the co-product credit are treated as explicit variables. Four research questions are addressed. RQ1: Do torrefied spent coffee grounds, spent tea leaves and nutshells reduce cradle-to-grave greenhouse-gas emissions when they replace lignite, under a convention that charges them with their upstream chain? RQ2: How do the treatment temperature and a fuel-quality constraint on it affect the net balance? RQ3: How far do supply-chain parameters, namely the logistics of the place of origin, the as-received moisture and the waste ratio, change the outcome? RQ4: To what extent does the conclusion depend on the allocation convention and on the scope of the co-product credit?

## 2. Materials and Methods

The assessment follows the principles and framework of ISO 14040 and ISO 14044 [32,33] and the product carbon-footprint requirements of ISO 14067 [34]. It is a product carbon footprint: It applies the life cycle framework to a single impact category, climate change, and is not a full life cycle assessment. Characterisation factors are the 100-year global warming potentials of the IPCC Sixth Assessment Report [35], and all results are expressed in kg CO_2_-eq. Foreground fuel properties are primary measurements (Section 2.2); background data on farming, transport, energy carriers and reference fuels are taken from Ecoinvent 3.10 [36] and from the sources listed in Section 2.1 and Section 2.6. No commercial LCA software was used: the model is a spreadsheet implementation of Equations (1)–(10), with background emission factors entered directly and the uncertainty propagation of Equation (9) computed in the same workbook, which is provided in full in the Supplementary Materials.

GenAI was used when preparing this article, model Claude for Mac, Claude 2.9939.2 (d3e504) 2026-09-24T17:20:35.000Z. Scope of genAI used was as follows: (1) data mining of price of materials, allowances, price of transport and transport distances (+sources); (2) visual generation of graphs; and (3) article copy review and fixing typos and semantics.

### 2.1. Feedstocks and Their Agricultural Context

Six materials were assessed. Five are food-processing residues: spent coffee grounds (SCGs), spent tea leaves (TEAs), and walnut (WAL), pistachio (PIS) and peanut (PEA) shells. The sixth, spruce wood chips (SPR) from bark-beetle calamity timber, is included as a non-food benchmark: it carries almost no agricultural burden and has no food co-product, so it isolates the effect of the food-credit mechanism described in Section 2.4. In the modelled supply chain the three nutshells are process streams of shelling plants, and SCG and TEA, the insoluble fractions left after brewing, are collected from cafés and catering; the beverage residues therefore arise in Europe rather than where the crop is grown, and what crosses the ocean is the green bean and the made tea. This provenance governs both the waste ratio of Table 1 and the as-received moisture of Section 2.6.

Each residue is generated in fixed proportion to a primary food commodity, and it is that commodity, not the residue, that drives the agricultural burden. Table 1 collects the waste ratio R (kg of waste per kg of primary product), the farming carbon footprint of the primary product, and the post-farm processing emissions associated with its primary use (coffee roasting and brewing, tea processing, nut cracking and sorting, and wood chipping). The farming carbon footprint per kilogram of waste follows from Equation (1):

CF_farm,waste_ = CF_farm,primary_/R (1)

where CF_farm,waste_ (kg CO_2_-eq kg^−1^) is the farming burden expressed per kilogram of waste biomass, CF_farm,primary_ (kg CO_2_-eq kg^−1^) is the published farming carbon footprint of the primary food commodity, and R (kg kg^−1^) is the waste-to-primary mass ratio.

The inverse dependence on R is important: A low waste ratio concentrates the farming burden of the crop onto a small mass of residue. Nutshells, at R = 0.35–0.50, concentrate their crop burdens by a factor of only two to three. For the beverage residues, R follows from what roasting and brewing remove: About 16% of the green bean is lost on roasting and a fifth to a quarter of the remainder is extracted, giving about 0.65 kg of dry grounds per kilogram of green coffee [1]; infusion dissolves roughly 30% of the dry tea leaf, giving about 0.70 kg of spent leaves per kilogram of made tea. Because R enters as an inverse multiplier on both the agricultural and the transport charge, it is among the most influential inputs of the model and is varied in Section 3.6.

### 2.2. Fuel Characterisation and Analytical Precision

The thermochemical characterisation is a primary measurement campaign carried out in 2026 on all six materials; it supersedes the values compiled in our earlier synthesis [19]. The five food residues were collected in several households over the course of 2025, and the samples of each material were pooled and homogenised into one composite sample; SPR is the composite sample of spruce wood chips described in [19]. Samples were milled to 1 mm using Retsch SM100 (Retsch GmbH, Haan, Germany) and treated for 30 min at 250, 300, 350, 450 and 550 °C in a LECO TGA 701 (LECO Corporation, St. Joseph, MI, USA) under an inert atmosphere, with the raw material retained as the reference state. Carbon, hydrogen, nitrogen and sulphur were determined on a LECO CHN628+S (LECO Corporation, St. Joseph, MI, USA), with oxygen by difference; ash and moisture were determined thermogravimetrically; the gross calorific value was measured on a LECO AC-600 (LECO Corporation, St. Joseph, MI, USA) isoperibolic calorimeter calibrated with benzoic acid. Mass yield was determined gravimetrically on the same treatment charges. Every method was applied in at least five replicates, and the variance of each replicate set was retained so that it could be carried through the model rather than quoted only in the characterisation table.

The net calorific value in the dry state was derived from the gross value by Equation (2), which subtracts the latent heat of the water formed from fuel hydrogen and of the residual analytical moisture:

NCV = GCV − 2.442 · (9 · H/100 + M/100)(2)

where NCV (MJ kg^−1^) is the net calorific value, GCV (MJ kg^−1^) is the gross calorific value, H (%) is the hydrogen content on a dry basis, M (%) is the analytical moisture, 2.442 (MJ kg^−1^) is the latent heat of vaporisation of water at 25 °C, and the factor 9 is the mass of water formed per unit mass of hydrogen.

Because the whole carbon balance is driven by the calorific value and the mass yield, treating these as point values would misstate the precision of the result. Section 2.8 sets out how the measured variances are propagated; every quantity in Section 3 that depends on them is reported with its standard deviation.

### 2.3. Goal, Scope, Functional Unit and System Boundary

This study has three objectives: (i) to quantify the net carbon footprint of each residue across the full treatment series, 250–550 °C; (ii) to determine, for each material, the treatment temperature that meets fuel-quality requirements for co-milling and co-firing with coal and report its carbon footprint; and (iii) to quantify the trade-off between the two—that is, the carbon saving forgone by imposing the fuel-quality constraint. The functional unit is one tonne of dry waste biomass entering the chain; the results are also reported per megajoule of fuel energy delivered so that materials of different energy density can be compared directly. Conversion and combustion are located in the Czech Republic, where the displaced fuel is lignite; the agricultural and logistics stages are located in each commodity’s principal producing regions (Section 2.6).

**Figure 1:**
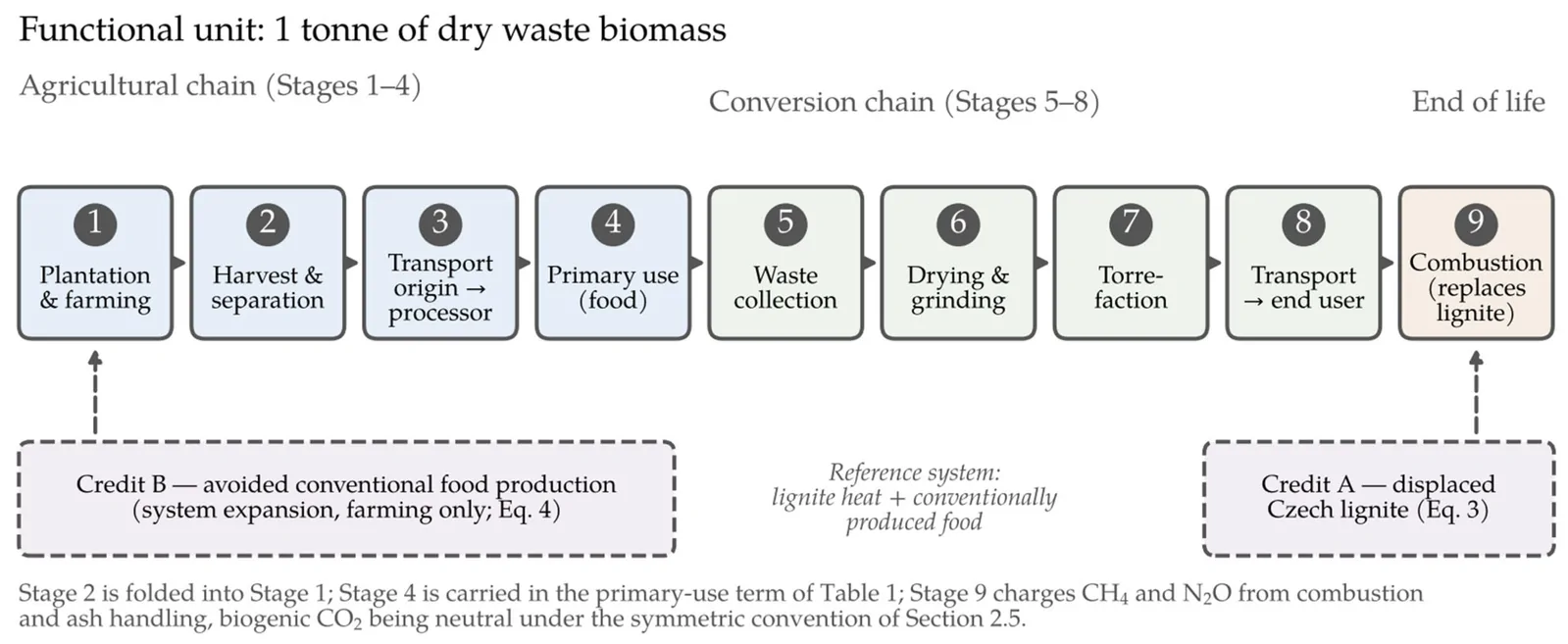
System boundary: nine process stages from cultivation to combustion, the two system credits of the fuel-first allocation and the reference system they represent. The functional unit is one tonne of dry waste biomass.

The system boundary is cradle-to-grave and comprises nine stages, as shown in Figure 1: (1) plantation and farming; (2) harvest and waste separation; (3) transport from the place of origin to the processor; (4) primary use, which is roasting and brewing, tea processing or shelling; (5) waste collection; (6) drying and grinding; (7) torrefaction; (8) transport of the torrefied product to the end user; and (9) combustion in place of lignite. Two system credits sit outside the linear chain and are described in Section 2.4. Stage 2 is folded into Stage 1, because the available farming inventories do not resolve harvest separately, and Stage 4 is set to zero because its emissions are already in the primary-use term of Table 1. For SCG and TEA, the packaging of the coffee and tea and the disposal of bags, wrappings and capsules are attributed to the food product and are not charged to the residue; the containers used to collect the residues at cafés and catering outlets fall within the Stage 5 collection factor, and their contribution was considered negligible. The TEA sample combined loose-leaf and bagged tea, with the bag material removed before analyses; the polymer fraction of tea bags and the aluminium and polymer of coffee capsules would add fossil emissions at combustion and are not part of the modelled residue, and capsule coffee residues are excluded. The grave of the system is Stage 9: combustion of the torrefied fuel, in which biogenic CO_2_ is not charged (Section 2.5) but CH_4_ and N_2_O are, followed by transport and landfilling of the ash; application of the solid to soil is not modelled.

A cradle-to-grave boundary was retained in preference to the gate-to-grave (cut-off) convention more common in waste-fuel studies, because cut-off would remove Stages 1 to 4 and, with them, the conservative character of the assessment; it is reported instead as scenario A (Section 2.10).

### 2.4. Fuel-First Allocation and System Expansion

Waste biomass is conventionally granted a zero upstream burden on the argument that it would have arisen regardless of any energy use. This convention hides the agricultural emissions entirely and makes any waste fuel look favourable by construction. We adopt the opposite and far more demanding convention, which we term fuel-first allocation: the waste biomass is treated as the primary product of the whole agricultural chain and carries 100% of the farming burden, while the food commodity is demoted to a co-product.

ISO 14044 sets a hierarchy for such multifunctional processes: Allocation should be avoided by subdivision or system expansion; where it cannot be, inputs and outputs are partitioned by physical relationships and, failing that, by other relationships such as economic value [33]. Waste-to-energy assessments most often apply the cut-off convention, which is also the regulatory default: The Renewable Energy Directive treats agricultural residues, including husks and nut shells, as having zero life cycle emissions up to the point of collection [42]. A consequential assessment would reach the same upstream result by a different route, because a residue is a dependent co-product for which its use does not change the volume of food produced; it would instead credit or charge the residue with its displaced alternative fate, landfill, composting or anaerobic digestion. Fuel-first allocation with system expansion is adopted here as a deliberately demanding attributional alternative, and Section 3.5 places it between the cut-off and economic conventions on one side and mass allocation on the other.

Consistency then requires that the food co-product be credited. Following the system-expansion (substitution) route recommended in ISO 14044 [33], the system is credited with the burden that would otherwise be incurred by producing the same quantity of food conventionally. Two credits therefore enter the balance. Credit A is the fossil emission avoided at the point of combustion by displacing lignite of equivalent energy content (Equation (3)). Credit B is the avoided conventional production of the food co-product (Equation (4)):

CreditA = −NCV_torr_ · (Y_m_/100) · EF_coal_ · 1000 (3)

where CreditA (kg CO_2_-eq t^−1^ waste) is the coal-displacement credit; NCV_torr_ (MJ kg^−1^) is the net calorific value of the torrefied product in the dry state; Y_m_ (%) is the torrefaction mass yield; EF_coal_ (kg CO_2_-eq MJ^−1^) is the emission factor of the reference lignite, CO_2_ plus the CH_4_ and N_2_O of its combustion; and the factor 1000 converts from kilograms to tonnes of feedstock. Lignite is displaced on an equal fuel-energy basis, which assumes the same boiler efficiency for both fuels; a lower relative efficiency of the torrefied fuel reduces Credit A in proportion and is examined in Section 3.6:

CreditB = −CF_farm,waste_ · 1000 (4)

where CreditB (kg CO_2_-eq t^−1^ waste) is the system-expansion credit for the food co-product, and CF_farm,waste_ (kg CO_2_-eq kg^−1^) is defined in Equation (1). CreditB is set to zero for SPR, which has no food co-product.

The product system thus delivers two functions per functional unit: E_r_ megajoules of solid fuel to a boiler and 1/R tonnes of the primary food commodity. The reference system delivers the same two functions conventionally: the same fuel energy from lignite and the same quantity of the same commodity from its usual supply chain (Figure 1). The substitution is assumed to be one-to-one within the same market, so the avoided food production carries the farming footprint of the food produced in the product system. This is an attributional system expansion used to keep the accounting of a deliberately reversed allocation self-consistent; it is not a consequential model of market responses.

The scope of CreditB requires an explicit decision that the literature usually leaves implicit. The credit applied here is restricted to the avoided agricultural production of the food co-product and is not extended to the avoided logistics of supplying that commodity to the European market, so it is a conservative lower bound; the symmetric variant is quantified in Section 3.5.

The energy of preparing the beverage, heating some 14 L of water per kilogram of roasted coffee and some 90 L per kilogram of tea, is not charged to the residue: the beverage is the co-product that Credit B already covers, and charging its preparation while crediting only the avoided farming would load one side of the expansion and not the other.

Two consequences of this formulation should be stated. First, because Credit B is constructed from the same farming footprint that enters Stage 1, the two cancel by construction; this is a property of the accounting convention, not an empirical result. Under one-to-one substitution, fuel-first allocation therefore charges the residue with exactly two upstream terms beyond the cut-off convention—the post-farm primary-use processing and the logistics of the primary product—and the base case equals cut-off plus these two terms (Section 3.5). Second, Credit B credits avoided industrial farming emissions (fertiliser-related N_2_O, diesel combustion and agrochemical manufacture) and not carbon sequestration; it must not be read as a removal.

The net carbon footprint is the sum of the nine process stages and the two credits, as described in Equation (5):

CF_net_ = ΣCF_i_ (i = 1…9) + CreditA + CreditB (5)

where CF_net_ (kg CO_2_-eq t^−1^ waste) is the net cradle-to-grave carbon footprint, and CF_i_ (kg CO_2_-eq t^−1^ waste) is the emission of process stage i. A negative CF_net_ denotes a net climate benefit.

### 2.5. Biogenic Carbon

Biogenic CO_2_ is treated as climate-neutral in accordance with the IPCC Guidelines [43]. The convention is applied symmetrically: the photosynthetic uptake of roughly 1.6–2.0 kg CO_2_ per kilogram of dry biomass during plant growth is not credited in Stage 1, and the corresponding release at combustion is not charged in Stage 9. Because the two are equal and opposite, the neutral convention is exact rather than approximate, and Stage 1 contains fossil and industrial emissions only. Biogenic neutrality applies to CO_2_ only: CH_4_ and N_2_O formed in combustion are charged in Stage 9 at the IPCC Tier 1 defaults for wood and wood waste, 30 and 4 kg TJ^−1^ [44], together with the transport and landfilling of the ash. The negative balances reported here are therefore avoided fossil emissions, not carbon removals; a permanent biogenic sink would arise only if the torrefied material were applied to soil instead of combusted, a route we do not model but return to in Section 3.10.

### 2.6. Inventory

Table 2 lists the process parameters and emission factors of the foreground system. The inventory departs from usual practice in two respects: the transport chain and the drying energy.

Transport. Stage 3 is built as a multi-leg chain from the usual place of origin: road collection within the producing region, deep-sea container freight to Hamburg or Rotterdam, and electric rail to the Czech Republic, with the emission factors of Table 2 [36,45,46] and the multi-leg accounting of EcoTransIT World [49]. WAL, which reaches the Czech market predominantly from France, Romania, Moldova and Ukraine, is modelled as road-only over 1500 km, and SPR is modelled as domestic haulage over 100 km. Country-of-origin shares are taken from the European Coffee Report for green coffee [50] and from FAOSTAT and Eurostat Comext trade data for tea and nuts [3,51]; Table 3 gives the resulting routes. Consistent with the fuel-first perspective, this leg is charged on the mass of primary product attributable to one tonne of residue, 1/R tonnes—for example, 1.54 t of green coffee per tonne of SCG. The nuts are taken to be transported in their shells and are shelled in Europe, so the shells cross the sea inside the nut. Stage 8, the delivery of the torrefied product to the end user, is 100 km of road freight scaled by the mass yield.

Drying. A single generic figure of the kind usually quoted, 0.27 MJ kg^−1^, cannot distinguish a sun-dried nutshell from fresh wood chips. Drying is therefore computed from the water actually evaporated per kilogram of dry solids, as described by Equation (6), at 3.0 MJ per kilogram of water for a conventional convective dryer, with 1.0 MJ kg^−1^ for advanced drying as a sensitivity [48]:

E_dry_ = h_dry_ · [m/(1 − m) − mt/(1 − mt)] (6)

where E_dry_ (MJ kg^−1^ dry solids) is the drying energy, h_dry_ (MJ kg^−1^ water) is the specific heat requirement of the dryer, m (kg kg^−1^, wet basis) is the as-received moisture of the feedstock, and mt (kg kg^−1^, wet basis) is the target moisture after drying, which is 0.05 here. Drying heat is supplied by natural gas at 0.202 kg CO_2_-eq MJ^−1^; grinding (0.12 MJ kg^−1^) and torrefaction (0.25 MJ kg^−1^) are electric and are charged at a Czech grid intensity of 0.38 kg CO_2_-eq kWh^−1^, slightly above the national emission factor of 0.37 for 2023 [47].

One rule governs the received moisture of all six materials: Each feedstock enters the inventory in the state in which it is handed over to the fuel supply chain, and water removed before that hand-over lies outside the boundary. The five food residues were sun-dried before they reached the laboratory, so the moisture determined on the characterised samples, 6.6 to 8.3%, is taken as the received moisture of a stream that is dried before hand-over. Spruce is the one material for which its laboratory sample was dried beyond its field state, and it is entered at an assumed 30%, the usual figure for roundwood seasoned at the landing over one summer before chipping. Because solar drying decides the result for the beverage residues, SCG and TEA collected wet are reported alongside the base case in Table 4, and fresh green chips are reported as a sensitivity case (Section 3.6). Table 4 gives the resulting water loads and drying energies.

The reference fuel is Czech lignite at 0.101 kg CO_2_ MJ^−1^, the IPCC Tier 1 default for lignite on a net-calorific-value basis, which is 101,000 kg CO_2_ TJ^−1^ with a 95% interval of 90,900–115,000 [44]; adding the CH_4_ and N_2_O of its combustion gives 101.4 g CO_2_-eq MJ^−1^. One fuel is used throughout for the carbon and the cost accounts alike: per unit of fuel energy, 0.101 kg CO_2_ MJ^−1^ is 0.364 t CO_2_ MWh^−1^, the figure that prices the allowances in Section 2.9. The hard-coal default of 0.0946 kg CO_2_ MJ^−1^ is carried as a sensitivity case. The fuel energy delivered per tonne of residue, which sets the magnitude of Credit A, is the energy residual of Equation (7):

Er = NCV_torr_ · Y_m_/100 (7)

where Er (MJ kg^−1^ of original dry biomass) is the energy residual, NCV_torr_ (MJ kg^−1^) is the net calorific value of the torrefied product, and Y_m_ (%) is the mass yield.

The carbon intensity of the delivered fuel, which allows a like-for-like comparison with coal, is given by Equation (8):

I = CF_res_/E_r_ (8)

where I (g CO_2_-eq MJ^−1^) is the carbon intensity of the fuel delivered to the end user, CF_res_ (kg CO_2_-eq t^−1^ waste) is the residual process burden—the sum of the nine stages after Credit B has cancelled the farming term but before Credit A is applied—and E_r_ (MJ kg^−1^) is the energy residual of Equation (7); the numerical factors of 1000 in the two units cancel.

### 2.7. Selection of the Treatment Temperature

For objective (ii), one treatment temperature was selected per material on fuel-quality grounds; objective (i) is addressed across the whole series in Section 3.3. The selection cannot be made on the carbon balance, because the energy residual and hence Credit A fall monotonically with temperature for all six materials, so the carbon balance alone would always choose the mildest treatment examined. The product must instead be hydrophobic, friable and compositionally close enough to coal to be co-milled and co-fired in existing plant, which requires the atomic ratios to have moved appreciably toward those of lignite [52,53]. The material-specific set-points of our earlier synthesis [19] are retained for comparability: 300 °C for SCG, WAL, PEA and SPR; and 250 °C for TEA and PIS. The new measurements support the two exceptions independently: At 300 °C, PIS loses 38.5% of the yield retained at 250 °C against 13.0% for SPR, and the ash content of TEA rises from 5.31 to 7.01%, the wrong direction for a fuel already at the top of the ash range (Section 3.9); holding TEA at 250 °C also preserves 325 kg CO_2_-eq t^−1^ of its savings. Hydrophobicity and grindability, which also bear on co-milling, were not determined in this campaign; the selection rests on the measured mass yield, ash and atomic ratios and on the optima reported in [17,18,19].

Molar H/C and O/C ratios are reported against the European Biochar Certificate criteria, H/C < 0.7, which is binding, and O/C < 0.4, which is recommended [54]. None of the six selected products meets them. The materials treated at 250–300 °C are therefore torrefied solid fuels, and the term biochar is reserved here for the hotter products that satisfy the following: 450 °C and above for four of the materials and 350 °C and above for WAL and PIS.

### 2.8. Uncertainty Propagation

Because the fuel properties are primary measurements with replicated determinations, their dispersion can be carried through the model instead of being quoted only in the characterisation table. Standard deviations were obtained from the replicate variances of carbon, hydrogen, nitrogen, ash, moisture, gross calorific value and mass yield, and they were propagated by the first-order law of Equation (9) in accordance with the Guide to the Expression of Uncertainty in Measurement [55]:

u(f)^2^ = Σ(∂f/∂xi)^2^ · u(xi)^2^ (9)

where u(f) is the standard uncertainty of a derived quantity f, xi denotes the measured inputs, and u(xi) denotes their standard uncertainties. Oxygen is obtained by difference, so its standard deviation is the quadratic sum of those of the measured fractions. The inputs are treated as mutually independent; since the calorimetric and gravimetric determinations share the same treatment charges, a positive correlation cannot be excluded, and it would make the combined uncertainty smaller than reported. The values are therefore a conservative bound.

The propagation reaches the net carbon footprint mainly through Credit A and, far more weakly, through Stage 8, for which its freight is charged on the surviving mass; these are the only terms in Equation (5) that depend on the measurement. The rest are driven by parameters and distances for which their uncertainty is addressed by the scenario analysis in Section 2.10.

### 2.9. Economic Assessment

Two economic quantities are derived. The first is the value of the avoided emissions under the EU ETS (Equation (10)) evaluated at five allowance prices: 50 EUR tCO_2_^−1^, close to the 2024 low; 65 EUR, the 2024 average; 84 EUR, the 2023 average [56]; and two illustrative long-term prices of 150 and 250 EUR:

VETS = |CF_net_| · pETS · 1000^−1^ (10)

where VETS (EUR t^−1^ waste) is the value of the avoided emissions, CF_net_ (kg CO_2_-eq t^−1^ waste) is the net carbon footprint from Equation (5), and pETS (EUR tCO_2_^−1^) is the allowance price.

The second is the delivered cost of energy, compared against a Czech brown-coal benchmark of 110 EUR t^−1^ [19] at a net calorific value of 13.9 MJ kg^−1^, within the range of Czech mined brown coal [57], equivalent to 28.49 EUR MWh^−1^; the 2024 average allowance price of 65 EUR tCO_2_^−1^ [56] at the lignite intensity of 0.364 t CO_2_ MWh^−1^ adds 23.63 EUR MWh^−1^ for a total of 52.12 EUR MWh^−1^. Feedstock costs are 30 EUR t^−1^ for SCG and TEA, 50 EUR t^−1^ for the three nutshells and 110 EUR t^−1^ for spruce chips; they are converted through the mass yield and the net calorific value to EUR per MWh of fuel energy. The comparison covers feedstock cost only; it excludes the capital and operating cost of the conversion plant, which Section 3.8 treats as the budget the difference must cover.

### 2.10. Sensitivity and Scenario Analysis

Three complementary analyses were performed. The first is a one-at-a-time parametric sensitivity in which each of the following parameters was varied across a plausible range while all others were held at their base values:

- The lignite emission factor, 0.0909–0.115 kg CO_2_ MJ^−1^, the IPCC 95% interval, with the hard-coal default of 0.0946 kg CO_2_ MJ^−1^ reported separately;
- The sea distance, ±30%, and the mode of the port-to-plant leg, electric rail or road haulage;
- The grid carbon intensity, 0.05–0.55 kg CO_2_ kWh^−1^, from a fully renewable supply to a coal-heavy grid;
- The specific drying requirement, 1.0–3.0 MJ per kilogram of water;
- The as-received moisture, ±10 percentage points, with the undried beverage residues and green spruce chips reported separately;
- The waste ratio R, ±30%, with the instant-coffee yield of 0.45 reported separately for SCG;
- The torrefaction energy, 0.10–0.45 MJ kg^−1^, with the lower bound representing an exothermic, autothermal process;
- The relative boiler efficiency of the torrefied fuel, 0.95–1.00;
- The farming carbon footprint, ±30%.

Separately, because allocation is the dominant methodological choice in waste-fuel assessments [27,58,59], five allocation conventions were compared: (A) cut-off, in which the residue enters the system burden-free at the point of discard, the regulatory default for agricultural residues [42]; (B) fuel-first with system expansion, the base case of Section 2.4; (C) fuel-first without any co-product credit, which charges the agricultural chain twice over by ignoring the food it produces and is not a defensible accounting choice, but bounds the result from below; (D) mass allocation, in which the upstream chain (Stages 1 and 3) is shared between residue and food in proportion to dry mass; and (E) economic allocation, in proportion to market value. Mass shares are the waste ratio R for the nutshells, 0.65/0.84 for SCG relative to the roasted bean and 0.70 for TEA relative to the made tea; value shares follow from residue prices of 30–50 EUR t^−1^ against 2024 commodity prices of 1150–4600 EUR t^−1^ [60,61,62] and remain below 5% even if those prices were three times lower. Finally, the scope of Credit B was varied between the restricted variant of the base case and the fully symmetric variant that also credits the avoided logistics of delivering the food commodity to Europe, labelled B2 and B1, respectively.

## 3. Results and Discussion

### 3.1. Fuel Properties and Atomic Ratios

**Figure 2:**
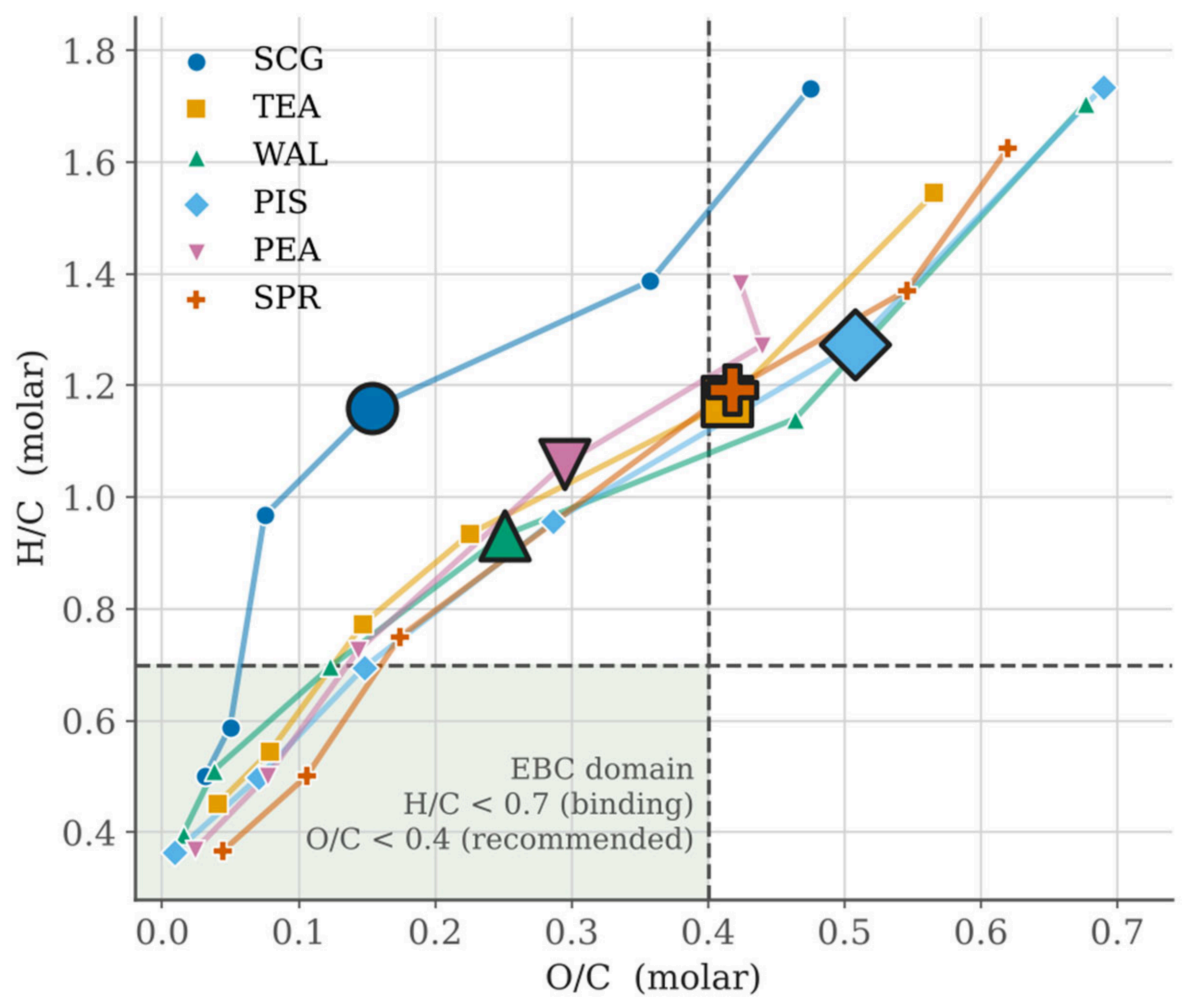
Van Krevelen diagram of all six materials in the raw state and at five treatment temperatures. Large, outlined symbols mark the temperature selected for each material; those of TEA and SPR nearly coincide. The shaded corner is the European Biochar Certificate domain, H/C < 0.7 and O/C < 0.4 [54].

Table 5 reports the fuel properties measured at the treatment temperature selected for each material, each with the standard deviation propagated from the replicate variances, and Figure 2 places all thirty-six determinations on a Van Krevelen diagram against the European Biochar Certificate thresholds.

Torrefaction raises the net calorific value of every material, but by very different margins: from 19.95 to 29.39 ± 0.31 MJ kg^−1^ for SCG and from 17.13 to 25.34 ± 0.26 MJ kg^−1^ for WAL, against only 17.11 to 20.27 ± 0.22 MJ kg^−1^ for TEA and 18.27 to 21.26 ± 0.22 MJ kg^−1^ for SPR. What separates the two groups is how much mass the treatment destroys, not the treatment itself: At the same 300 °C, spruce retains 73.6 ± 0.7% of its mass compared with 52.3 ± 0.5% and 52.0 ± 0.5% for SCG and WAL, and the two materials that held at 250 °C, TEA and PIS, retain 78.3 ± 0.8% and 73.0 ± 0.7%. The energy residual, the quantity that drives the carbon balance, is far more uniform, spanning only 13.19 ± 0.19 to 15.87 ± 0.23 MJ kg^−1^ across all six materials, because the gain in calorific value and the loss of mass largely offset one another.

The atomic ratios in Figure 2 follow the classical carbonisation trajectory, and they place the selected products unambiguously outside the certified-biochar domain: H/C ranges from 0.932 ± 0.013 for WAL to 1.273 ± 0.018 for PIS and O/C from 0.154 ± 0.008 to 0.508 ± 0.009, against thresholds of 0.7 and 0.4. Only at 450 °C and above do all six materials satisfy both criteria. This is the empirical basis for the terminology of Section 2.7: At the temperatures that make carbon sense, these materials are torrefied solid fuels and describing them as biochar would misstate their regulatory status. Independent characterisation of nutshell chars as a solid fuel reports comparable atomic ratios and energy properties [63,64].

### 3.2. Stage-by-Stage Carbon Balance

**Figure 3:**
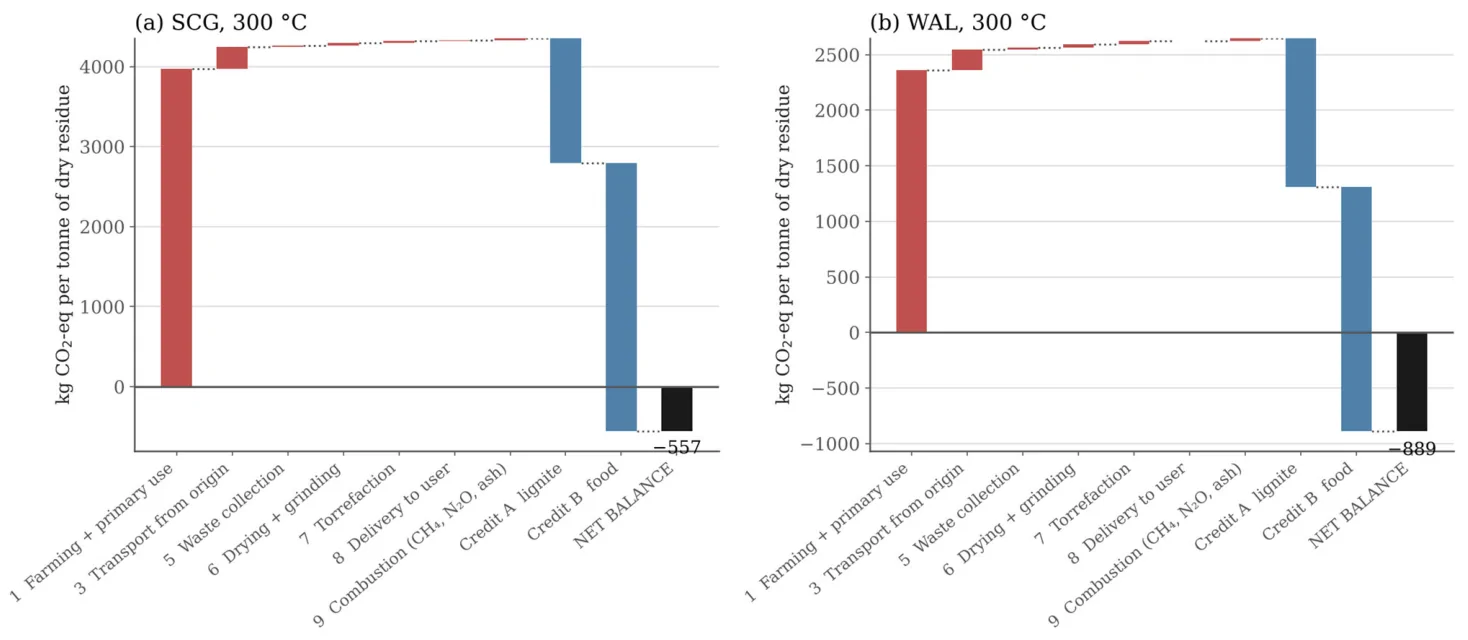
Stage-by-stage carbon balance for (**a**) SCG torrefied at 300 °C and (**b**) WAL torrefied at 300 °C. Positive bars are emissions; negative bars are credits; the final bar is the net cradle-to-grave carbon footprint. Note the difference in vertical scale between the two panels.

Table 6 gives the complete nine-stage inventory for all six materials at their selected temperature, and Figure 3 renders the balance as a waterfall for two contrasting cases: SCG, a material dominated by its agricultural burden, and WAL, a typical nutshell.

Before credits, the gross emissions of the five food residues are dominated by Stage 1. For SCG, the farming and primary-use term is 3969 kg CO_2_-eq t^−1^ against 386 kg for the whole process side, a ratio of 10 to 1, and for TEA, for which its crop carries the largest farming footprint of the set, the ratio is 41 to 1. SPR inverts this completely: its Stage 1 is only 50 kg CO_2_-eq t^−1^, while its process side reaches 322 kg, of which 228 is drying alone.

Stage 3, charged from the real place of origin and on the primary-product mass, is the largest process-side term of every food residue. It reaches 513 kg CO_2_-eq t^−1^ for PEA, 334 for PIS, 276 for SCG, 275 for TEA, and 186 for WAL against 6 kg CO_2_-eq t^−1^ for domestic SPR, two and a half to seven times the sum of collection, drying, grinding and torrefaction. Two features explain the magnitude: the 1/R multiplier, from 1.43 for TEA to 2.86 for PEA, and the length of the chain rather than its intensity. Deep-sea freight at 0.0127 kg CO_2_-eq t^−1^ km^−1^ is five times less carbon-intensive per tonne-kilometre than road haulage, but 12 000 km of it still dominates.

Applying the credits changes the picture entirely. Credit B removes the farming term exactly, leaving only primary-use processing as a residual agricultural charge: 615 kg CO_2_-eq t^−1^ for SCG, 214 for TEA, 160 for WAL, 200 for PIS and 171 for PEA. The residual process burden, everything that survives the cancellation, is therefore 1001, 592, 449, 632, 787 and, for SPR, which receives no credit at all, 372 kg CO_2_-eq t^−1^. At 101.4 g CO_2_-eq MJ^−1^ and an energy residual of 13.2 to 15.9 MJ kg^−1^, displacing lignite is worth −1338 kg CO_2_-eq t^−1^ (WAL) to −1610 (TEA). Every material closes negative, but the ratio of credit to residual burden runs from 4.3 for SPR down to 1.6 for SCG, so a few hundred kilograms anywhere in the chain matters.

Because Credit B is constructed from the same farming footprint as Stage 1, the two cancel by construction; this is a property of the accounting convention, not an empirical result. Its practical meaning is that, under one-to-one substitution of the food co-product, fuel-first allocation charges the residue with exactly two upstream terms beyond the cut-off convention, the post-farm primary-use processing and the logistics of the primary product; the base case therefore equals scenario A plus these two terms, and scenario B1 equals A plus the primary-use term alone. What the convention does show is which upstream terms a residue must carry if it is held responsible for its supply chain: the supply chain (Stages 3, 5 and 8) accounts for 46–68% of the residual burden of TEA and the three nutshells, the primary-use processing for 61% of that of SCG, and drying for 61% of that of SPR.

### 3.3. Influence of Treatment Temperature

**Figure 4:**
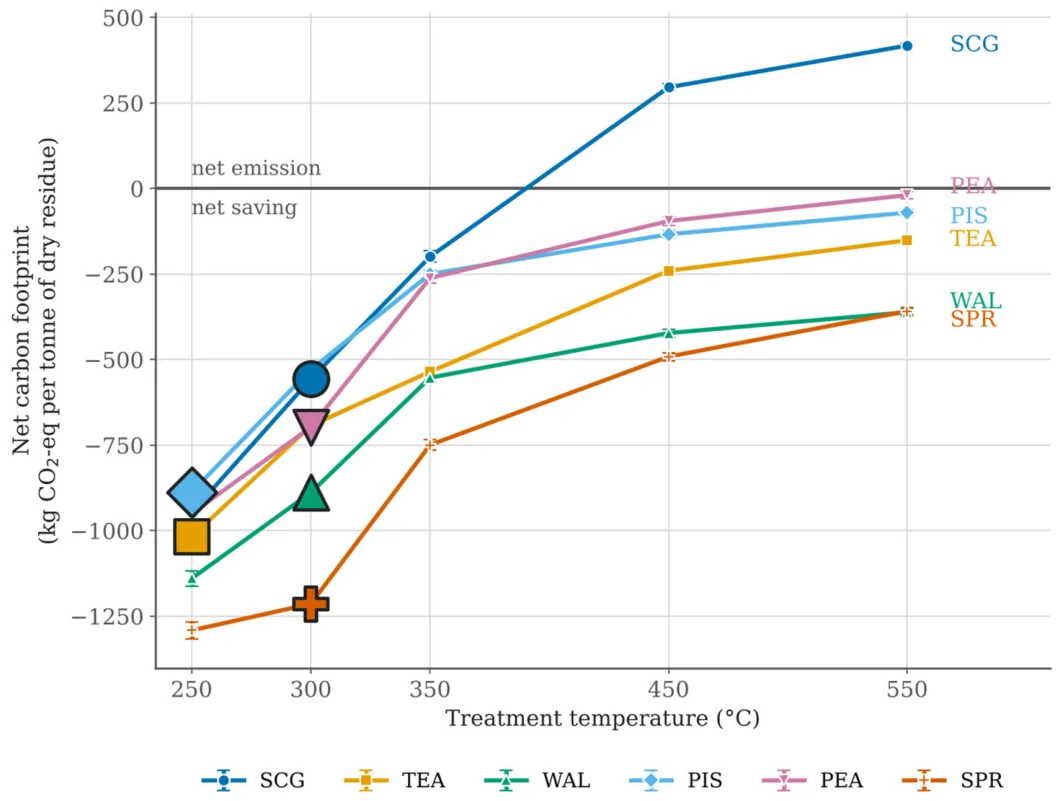
Net cradle-to-grave carbon footprint as a function of treatment temperature. Error bars are one standard deviation propagated from the replicate variances; large, outlined symbols mark the temperature selected for each material in Table 5. Values below zero denote a net carbon saving. The untreated raw state is omitted.

Figure 4 traces the net carbon footprint across the full treatment series, with the propagated measurement uncertainty as error bars. The relationship is monotonic for every material: the colder the treatment, the larger the saving. Only SCG crosses from saving to emission within the series between 350 and 450 °C, moving from −199 ± 17 to +296 ± 10 kg CO_2_-eq t^−1^. The other five remain negative throughout, with SPR ranging from −1292 ± 24 at 250 °C to −358 ± 10 kg CO_2_-eq t^−1^ at 550 °C and PIS and PEA only just at −70 ± 10 and −19 ± 11 kg CO_2_-eq t^−1^ at 550 °C.

The explanation lies in Equation (3): Credit A is directly proportional to the energy residual, and the energy residual declines monotonically with temperature because mass yield falls faster than calorific value rises. SCG illustrates the trade-off sharply. Raising the temperature from 250 to 350 °C lifts the net calorific value from 24.10 to 31.57 MJ kg^−1^, a gain of 31%, but cuts the mass yield from 80.1 to 37.2%, so the energy residual collapses from 19.30 to 11.75 MJ kg^−1^, and 39% of the recoverable energy—and with it 39% of the displaceable coal—is lost to the volatile stream.

The carbon balance alone therefore always favours the mildest treatment, and the optimum cannot be set from the carbon balance. It is fixed instead by fuel quality, as set out in Section 2.7, and the penalty this exacts (objective iii) is substantial. Moving from 250 to 300 °C costs 391 kg CO_2_-eq t^−1^ of savings for SCG, 41.2% of what the milder treatment would have delivered; 253 (26.7%) for PEA; 251 (22.1%) for WAL; and 78 (6.0%) for SPR. For TEA and PIS, it held at 250 °C, and the constraint costs nothing. This improvement is accompanied by a genuine change in the fuel: between 250 and 300 °C, the molar O/C ratio of SCG falls from 0.358 to 0.154, and that of WAL falls from 0.464 to 0.250, the range in which the materials acquire coal-like behaviour [65,66,67].

This qualifies the conclusion of Cho and Lee [9], who identified about 270 °C as optimal for co-firing torrefied residual biomass. On the carbon balance alone, the optimum lies at the lower bound of the torrefaction window for every material examined here, and any higher set-point is a fuel-quality decision that is legitimate but one that should be declared and costed, as shown in Figure 4, rather than presented as a joint optimum.

### 3.4. Net Carbon Balance and Carbon Intensity Relative to Coal

**Figure 5:**
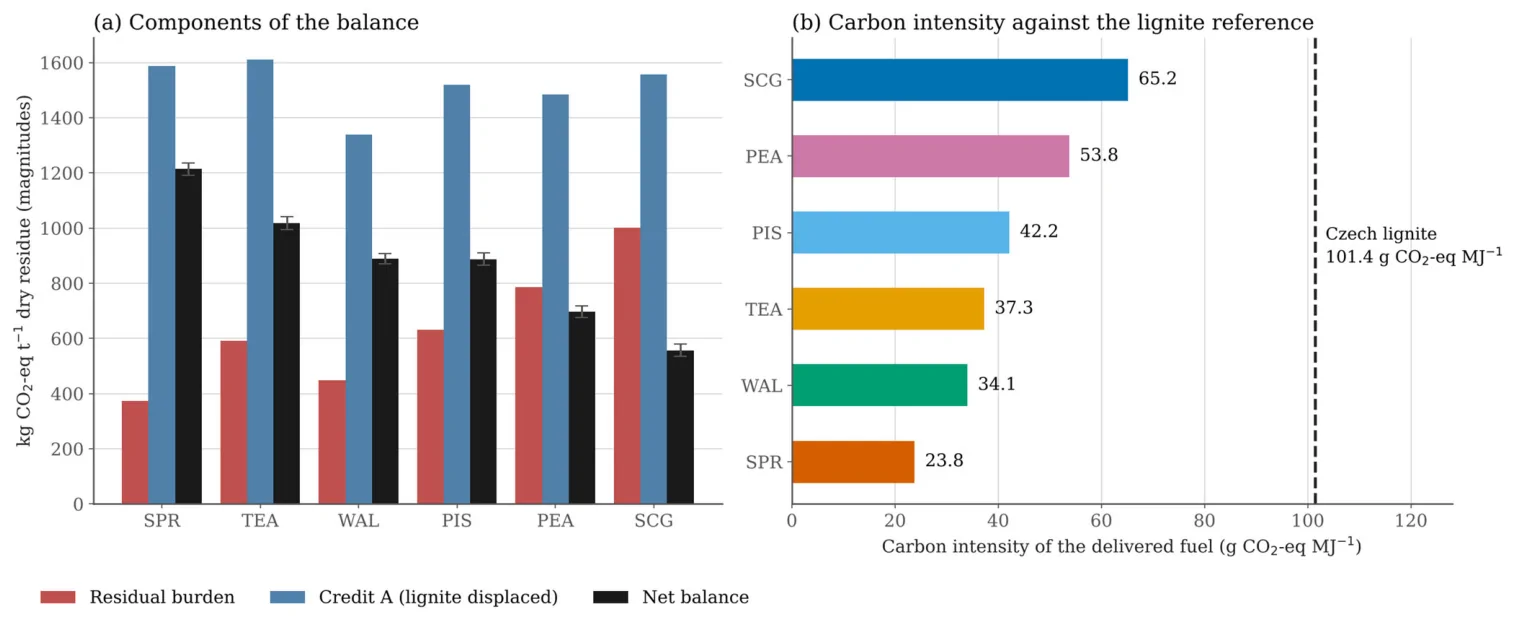
(**a**) Residual process burden, lignite-displacement credit and resulting net balance for each material at its selected temperature; all values are magnitudes, and the error bars apply to the net balance. (**b**) Carbon intensity of the delivered fuel compared with the Czech lignite reference of 101.4 g CO_2_-eq MJ^−1^; every material lies below it.

Table 7 and Figure 5 summarise the outcome at the selected temperatures. All six materials deliver a net saving: SPR (−1214 ± 23 kg CO_2_-eq t^−1^), TEA (−1018 ± 23), WAL (−889 ± 19), PIS (−887 ± 22), PEA (−697 ± 21) and SCG (−557 ± 22). The propagated measurement uncertainty is 1.9–3.0% of the net balance for five materials and 4.0% for SCG, for which its balance is the smallest; it is far smaller everywhere than the supply-chain assumptions examined in Section 3.6. Table 7 also gives SCG and TEA collected wet at 65 and 75% moisture, alongside the pre-dried base case: dried with natural gas, they become net emitters of +514 and +755 kg CO_2_-eq t^−1^, and their delivered fuel is 33 and 47% more carbon-intensive than the lignite it would replace.

Expressed as a carbon intensity of the fuel actually delivered, shown in Equation (8), the six materials span 23.8 g CO_2_-eq MJ^−1^ (SPR), 34.1 (WAL), 37.3 (TEA), 42.2 (PIS), 53.8 (PEA) and 65.2 g CO_2_-eq MJ^−1^ (SCG) against 101.4 g CO_2_-eq MJ^−1^ for the lignite reference, avoiding 76.5, 66.4, 63.2, 58.4, 47.0 and 35.7% of the emissions of the coal they replace. The two rankings agree at the top and bottom but not in the middle: TEA is second by absolute savings and third by intensity, and WAL exhibits the reverse, because TEA delivers more fuel energy per tonne (15.87 against 13.19 MJ kg^−1^) and therefore displaces more coal.

What neither ranking follows is the fuel. SCG leads the set on the two properties that drive the balance, the highest net calorific value at the selected temperature, 29.39 MJ kg^−1^, and an energy residual within 4% of the best material, yet it comes last. What holds it back is arithmetic rather than thermochemistry: the waste ratio multiplies both the primary-use term of Table 1 and the Stage 3 freight by 1/R, so 615 and 276 of the 1001 kg CO_2_-eq t^−1^ residual burden, 89% of it, are charges the material incurs for being a small fraction of a heavily traded crop rather than for anything it does in the furnace.

SPR is the mirror image and heads both rankings. It carries almost no agricultural burden, receives no food credit and has no maritime leg, so its residual burden of 372 kg CO_2_-eq t^−1^ is the smallest of the set even though 228 of it, three-fifths, is drying alone. What makes that figure small is a step that happens before the inventory begins: seasoning the roundwood at the landing over one summer, which costs nothing and is ordinary forestry practice. If chipped green at 50% moisture, the same wood would carry 574 kg CO_2_-eq t^−1^ of drying and save 868 rather than 1214, which would place it fourth, behind TEA, WAL and PIS.

These intensities are consistent with the cradle-to-gate literature once the difference in boundary is allowed for. Assessments of SCG pellets and of conventional and microwave torrefaction report roughly 7–10 g CO_2_-eq MJ^−1^ [68]; our 24 to 65 g CO_2_-eq MJ^−1^ additionally includes the primary-use stage and an intercontinental transport chain that gate-boundary studies exclude by construction. The full-chain assessment of sugarcane bagasse carbonisation reported about 1.2 t CO_2_-eq per tonne of feedstock [25], comparable with the top of the 0.56 to 1.21 t CO_2_-eq t^−1^ obtained here.

### 3.5. Allocation Convention and the Scope of the Food Credit

**Figure 6:**
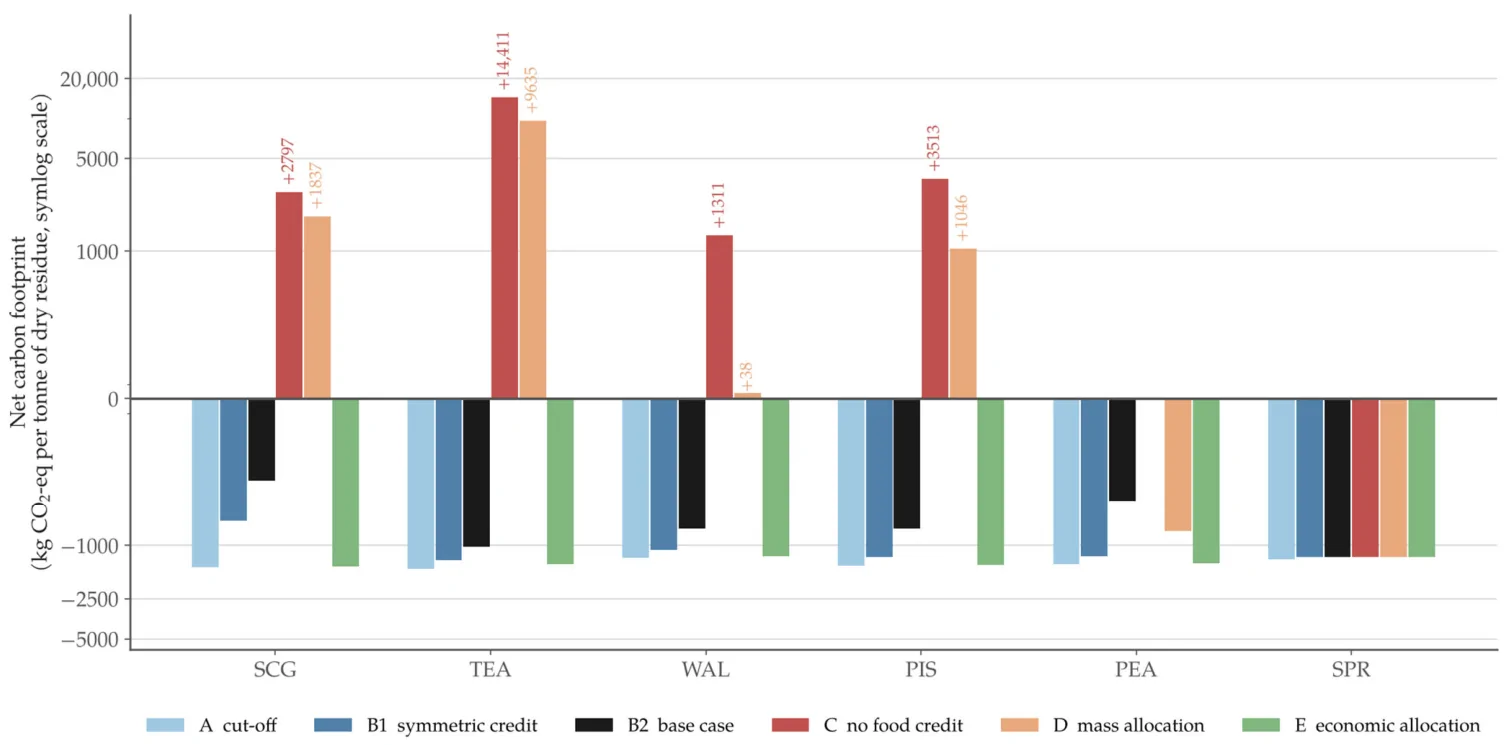
Net carbon footprint under six accounting conventions, on a symmetric logarithmic scale. Under Scenarios C and D, every food residue except PEA becomes a net emitter; the positive values are labelled. SPR, having no food co-product, is identical under B1, B2, C, D and E.

Table 8 and Figure 6 compare the accounting conventions, and the choice decides the sign of the result for almost every food residue. Under the cut-off treatment (A), in which the residue enters the system free of upstream burden, all six materials save between 1235 and 1507 kg CO_2_-eq t^−1^ and become almost indistinguishable, because all that remains is the conversion chain set against each material’s coal credit. That is the convention most published waste-fuel assessments adopt and the regulatory default [42]. Economic allocation (E) stays within 35 kg CO_2_-eq t^−1^ of it for four of the food residues and within 118 for TEA, because a residue worth 30–50 EUR t^−1^ carries less than 2% of the value of the crop it comes from.

The base case (B2) is far more demanding. It costs 4% of the cut-off saving for SPR, 28% for WAL, 32% for TEA, 38% for PIS, 50% for PEA and 62% for SCG; in every case, this reflects the primary-use and freight burden that the waste ratio amplifies.

Scenarios C and D are the decisive tests. Removing the food credit while retaining the full farming burden (C) turns four of the five food residues into net emitters, +14,411 kg CO_2_-eq t^−1^ for TEA, +3513 for PIS, +2797 for SCG and +1311 for WAL, and leaves PEA, for which its crop has the smallest farming footprint, at break-even (−11); SPR is unaffected at −1214 kg CO_2_-eq t^−1^. Mass allocation (D), which shares the upstream chain by dry mass and so charges a residue with a large part of a crop for which its production it does not cause, gives the same verdict for the same four: TEA (+9635), SCG (+1837), PIS (+1046) and WAL (+38) become net emitters, and only PEA retains a saving (−902 kg CO_2_-eq t^−1^). The results confirm, in a specific setting, the general warning of Matuštík et al. [27] that the carbon-negative label can hinge on a methodological decision. Only SPR, a dedicated energy feedstock with no co-product to argue about, is carbon-negative under every convention tested. For the five food residues, the saving holds under cut-off, economic allocation and system expansion; under mass allocation or without a food credit, it holds only for PEA. We regard system expansion as the self-consistent reading of ISO 14044 for a system that is charged for growing coffee, tea or nuts, and mass allocation is inappropriate here, because the residue does not drive the production of the crop and a physical partition should reflect a causal relationship; readers who take the opposite view should read the results under Scenario D.

The scope of Credit B is a second and finer methodological choice. Crediting the avoided logistics, as well as the avoided farming, the fully symmetric variant B1 improves every food residue: TEA moves from −1018 to −1293 kg CO_2_-eq t^−1^, PEA from −697 to −1210, PIS from −887 to −1221, WAL from −889 to −1075 and SCG from −557 to −833. SPR is unaffected, having no co-product. The symmetric variant is arguably the stricter reading of ISO 14044, since what the system displaces is food delivered to the European market and not a crop standing in a field in Brazil; we nevertheless report the restricted variant as the base case, because it flatters the result least.

### 3.6. Parametric Sensitivity and Measurement Uncertainty

**Figure 7:**
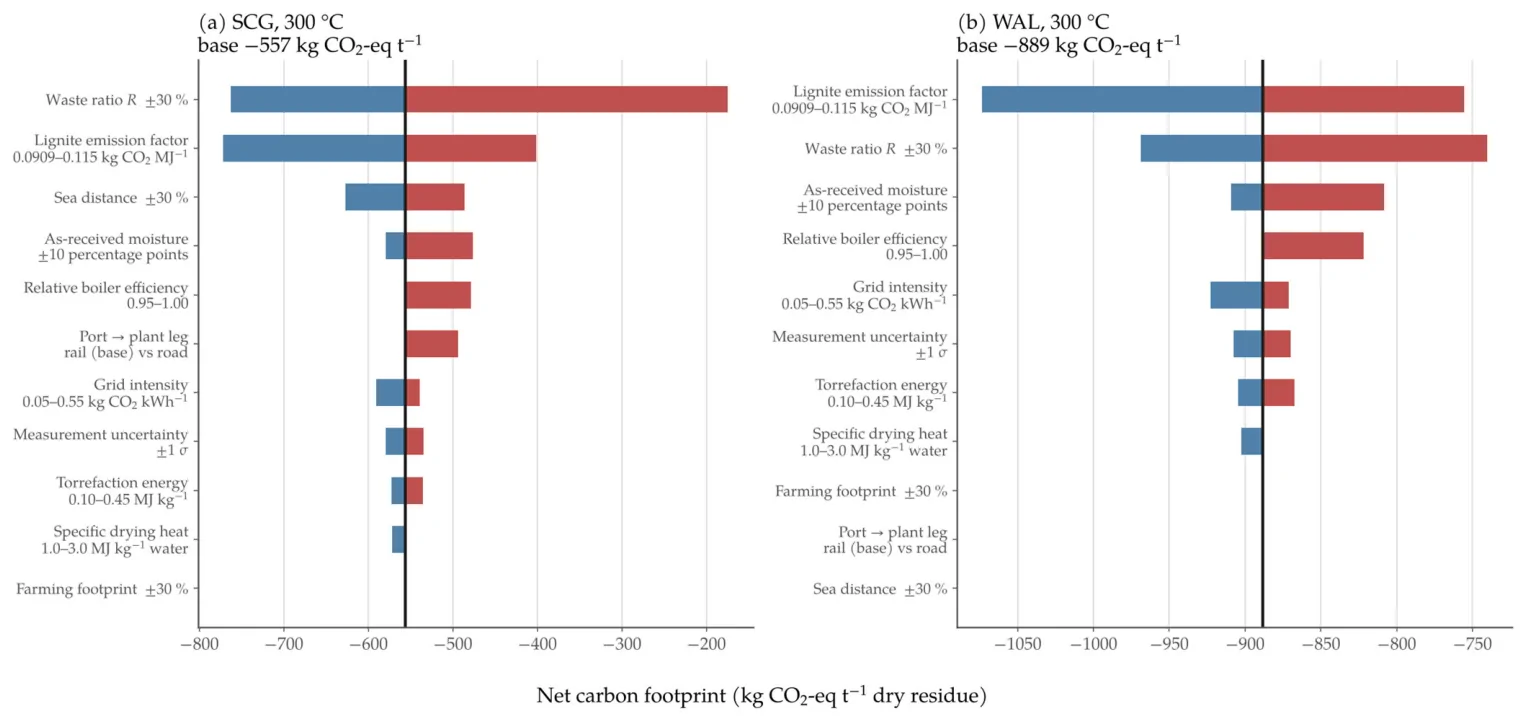
One-at-a-time sensitivity of the net carbon footprint for (**a**) SCG at 300 °C and (**b**) WAL at 300 °C. Blue bars move the balance toward a larger saving, red bars toward a smaller one, irrespective of which end of the parameter range produces them; the vertical line is the base case. The waste ratio is perturbed by ±30%, the as-received moisture by ±10 percentage points, floored at the 5% target, and the lignite emission factor across its IPCC 95% interval; the alternative feedstock states of Section 2.6 lie further out than the bars shown. The accounting conventions are treated separately in Figure 6.

Figure 7 shows the one-at-a-time sensitivity for SCG and WAL. Moving the emission factor of the displaced lignite across the IPCC 95% interval, 0.0909 to 0.115 kg CO_2_ MJ^−1^, shifts the net balance by 370 kg CO_2_-eq t^−1^ for SCG and 318 for WAL and by 353–382 for the other four materials; it is the largest conventional lever for every material. The hard-coal default of 0.0946 kg CO_2_ MJ^−1^ reduces every saving by 84 to 102 kg CO_2_-eq t^−1^ and changes no sign. A 5% lower boiler efficiency for the torrefied fuel than for lignite reduces Credit A in proportion by 67–81 kg CO_2_-eq t^−1^.

The transport parameters matter most where the chain is longest and the multiplier is largest. Varying the sea distance by ±30% moves PEA by ±131 kg CO_2_-eq t^−1^, 19% of its saving, SCG by ±70 (13%), PIS by ±84 (9%) and TEA by ±71 (7%); WAL and SPR have no maritime leg. Replacing electric rail with road haulage on the 800 km port-to-plant leg costs PEA 117 kg CO_2_-eq t^−1^, PIS 82, SCG 63 and TEA 59. Both changes together consume 36% of the savings of PEA and 24% of that of SCG, so the logistics assumptions bound the result without deciding its sign for any material.

Among the conversion parameters, drying matters chiefly for SPR: cutting the specific drying requirement from 3.0 to 1.0 MJ per kilogram of water is worth 8 to 15 kg CO_2_-eq t^−1^ for the sun-dried food residues but 152 for SPR, 13% of its net saving. Decarbonising the electricity supply from 0.55 to 0.05 kg CO_2_ kWh^−1^ is worth 51 kg CO_2_-eq t^−1^ (4–9%), and the full range of torrefaction energies is 37 kg CO_2_-eq t^−1^ (3–7%). The farming carbon footprint, varied by ±30%, has no effect on the food residues for the structural reason given in Section 3.2 and moves SPR, which receives no credit, by only ±9 kg CO_2_-eq t^−1^.

Two inputs that are neither fuel properties nor emission factors deserve separate treatment, because each can reverse a verdict on its own and neither is bounded by the laboratory. The first is the waste ratio. Varied by ±30%, it moves SCG between −763 and −175 kg CO_2_-eq t^−1^, a swing of 588 that no other single parameter approaches, because R is an inverse multiplier on two of the three largest positive terms at once. Taking instead the yield reported for industrial instant-coffee manufacture, R = 0.45, the saving falls to 160 kg CO_2_-eq t^−1^; at R = 0.20, the material becomes a net emitter of +1450 kg CO_2_-eq t^−1^ for which its delivered fuel is almost twice as carbon-intensive as the lignite it would displace. TEA is less exposed because its primary-use term is small: at R = 0.65, which is the ceiling of industrial aqueous extraction, it still saves 980 kg CO_2_-eq t^−1^.

The second is the received moisture. Both beverage residues leave the cup wet, with SCG at about 65% moisture and TEA at about 75%, because the leaf swells as it infuses. Charging the pathway by evaporating water at 3.0 MJ kg^−1^ adds 1071 kg CO_2_-eq t^−1^ to SCG and 1773 to TEA, turning savings of 557 and 1018 into emissions of 514 and 755. The solar drying assumed in Section 2.6 is therefore not a detail of the inventory but the condition on which both results rest, which is why Table 7 reports both states. Fresh spruce chips at 50% rather than seasoned chips at 30% cost 346 kg CO_2_-eq t^−1^, 28% of that material’s saving. None of these figures is a measurement uncertainty; all are supply-chain design choices, and they are where further work would be most valuable.

Measurement uncertainty is smaller than any of these. Propagating the replicate variances of the calorific value, hydrogen, moisture and mass yield through Credit A gives a standard deviation of the net balance of 19 to 23 kg CO_2_-eq t^−1^, split almost evenly between the calorific value (52–53%) and the mass yield (47–48%), with hydrogen and analytical moisture together below 0.2%. The characterisation is the best-constrained part of the assessment, and further effort belongs in the logistics and feedstock-state inventory and in the choice of counterfactual fuel.

### 3.7. Drying Energy and Autothermal Operation

Computing drying from the actual moisture load also yields a criterion for autothermal operation: whether the volatile stream released during torrefaction carries enough energy to dry the incoming feedstock [48]. Taking the volatile energy as the difference between the calorific value of the raw material and the energy residual of Equation (7), the five sun-dried food residues carry 18 to 41 times more energy in their volatiles than their drying requires. Seasoned SPR chips at 30% moisture need 1.13 MJ kg^−1^ against a volatile stream of 2.63 MJ kg^−1^, a coverage of 233%; at 50% moisture, they need 2.84 MJ kg^−1^, and the coverage falls to 92%, so a line on fresh calamity wood would fall short of self-sufficiency before heat-recovery losses are counted. Whether a torrefaction plant on woody feedstock is autothermal is therefore decided at the forest landing by a summer of seasoning rather than in the reactor.

### 3.8. Economic Assessment

Table 9 monetises the avoided emissions and compares delivered fuel costs. At the 2024 average allowance price of 65 EUR tCO_2_^−1^ [56], the avoided emissions are worth 79 EUR per tonne of SPR, 66 EUR for TEA, 58 EUR for WAL and PIS, 45 EUR for PEA and 36 EUR for SCG. Across the price range examined, 50 to 250 EUR tCO_2_^−1^, the value spans 28–139 EUR t^−1^ for SCG and 61–304 EUR t^−1^ for SPR.

On the fuel side, the torrefied products are competitive: at their selected temperatures, the material cost is 6.80 EUR MWh^−1^ for TEA, 7.03 for SCG, 12.01–13.65 for the nutshells and 25.32 for SPR compared with 52.12 EUR MWh^−1^ for brown coal once its allowance cost of 23.63 EUR MWh^−1^ is included. Expressed per tonne of residue, that advantage is 200 EUR for TEA, 192 for SCG, 167 for PIS, 162 for PEA, 141 for WAL and 116 for SPR.

The two measures are not additive: The 52.12 EUR MWh^−1^ benchmark already charges brown coal for its own allowances, so the fuel-cost advantage already contains the value of the combustion CO_2_ that the residue avoids, and adding the ETS value of the net balance would count that benefit twice. The fuel-cost advantage is the envelope a plant operator faces, and it exceeds the carbon value by a factor of 1.5 for SPR and 2.4–5.3 for the food residues, so for every material, the economic case rests on the fuel price rather than on the carbon price. Whether a drying, grinding and torrefaction line can be built and run within 116 to 200 EUR per tonne of throughput is a question for a techno-economic study of the kind performed for torrefaction and pyrolysis systems elsewhere [69,70,71,72], and it is not answered here. The binding constraint is more likely the absence of an organised collection market: the beverage residues are dispersed across cafés, catering outlets and households, and their notional 30 EUR t^−1^ price reflects an aggregation problem rather than a scarcity.

### 3.9. Ash, Slagging and Operational Suitability

Carbon performance and operational suitability are not the same property. TEA carries 5.31 ± 0.05% ash at 250 °C, rising to 7.01% at 300 °C and 11.75% at 550 °C as the organic matrix is progressively removed. WAL, at 4.54%, is the next highest; SPR at 0.71% and PIS at 1.46% are the cleanest. TEA also carries 2.37% nitrogen and SCG carries 3.39%, against 0.33% for SPR, which bears on fuel NO_x_ in any combustion system without staged air [73,74,75].

A quantitative slagging assessment is not possible from the present data, because the conventional indices require the elemental composition of the ash, which was not determined; such indices also transfer poorly from coal to biomass ashes, and laboratory ash-fusion temperatures are the more reliable route [76,77,78,79]. Ash-fusion determination is therefore the next measurement required.

The consequence for the recommendation is explicit. WAL, PIS, PEA and SPR can be recommended on both counts. TEA has the second-largest carbon saving of the set, but with ash above 5% and rising with severity, it should not be proposed for pulverised-fuel co-firing without ash-fusion testing, and it suits grate or fluidised-bed systems with controlled ash removal. SCG combines a low ash content, 2.96%, with the highest nitrogen content of the set, 3.39%; its combustion behaviour was not tested here, and co-firing tests of SCG–wood pellets report significantly higher NO emissions than for pure wood, though not in proportion to the SCG share [10]. Its carbon saving is also the smallest of the set and rests on the supply-chain assumptions of Section 3.6 rather than on its fuel properties, so it can be recommended only where the grounds reach the plant already dried. The high nitrogen contents of SCG (3.39%) and TEA (2.37%) will materially affect NO_x_ formation, as other studies report [73,74,75,80], and may in practice exclude both fuels from plants without selective catalytic reduction, whatever their carbon footprint.

### 3.10. Limitations

Eleven limitations qualify these results. First, the emission factor of the displaced lignite is among the largest levers on the outcome, and it is a choice as much as a measurement. The IPCC Tier 1 default carries a 95% interval of −10 to +14%, and the real counterfactual in a Czech boiler is a specific brown coal [81] for which its carbon content was not measured for this study. None of that range changes any sign, but it moves every balance by up to 222 kg CO_2_-eq t^−1^, two-fifths of the smallest saving in the set.

Second, the farming and primary-use inventories are literature values for globally distributed crops applied to a Czech conversion and combustion context (Table 1). The cancellation of Section 3.2 removes the farming term from the base-case result but not from Scenarios C and D, and the primary-use term survives in all of them; it is the least well-constrained input that still matters, particularly for SCG, where it accounts for 61% of the residual burden.

Third, the origin shares underlying the transport model are trade statistics rather than consignment-specific. Green coffee shares are taken from the European Coffee Report [50] and the remainder from FAOSTAT and Eurostat Comext trade matrices [3,51,82,83], which describe what Europe imports and not what any individual processor receives. The sensitivity analysis bounds the consequence.

Fourth, the credit-scope asymmetry described in Section 2.4 is a deliberate conservatism and not a neutral choice. Charging the delivery of the primary product without crediting the delivery of the food it displaces understates the benefit by 186 to 513 kg CO_2_-eq t^−1^, which for PEA is 74% of its base-case saving. Readers preferring the symmetric treatment should read the B1 column of Table 8.

Fifth, the assessment covers a single impact category. The ash and nitrogen contents of Section 3.9 raise questions of particulate emissions, NO_x_ formation, slagging and fouling that a carbon footprint does not address [84,85].

Sixth, the end-of-life route modelled here is combustion. If the torrefied solid were instead applied to soil, the photosynthetic uptake excluded under the symmetric biogenic convention of Section 2.5 would not be offset by combustion and would constitute a genuine long-term sink, at the cost of forgoing Credit A [86,87,88]. The trade-off is finely balanced, 1.3 to 1.6 t CO_2_-eq t^−1^ of avoided fossil emissions against roughly 0.5 t of carbon retained in a stable solid, and the growing soil-application literature [31] frames it without settling it. Our own phytotoxicity results [16,17] suggest the answer is feedstock-specific.

Seventh, the moisture convention of Section 2.6 places the sun-drying that precedes hand-over outside the boundary. This is defensible, since solar drying carries no emissions and the residues would be dried in any case for transport weight and against mould, but it is load-bearing: The convention is worth 1071 kg CO_2_-eq t^−1^ to SCG and 1773 to TEA, enough in both cases to turn a saving into an emission (Table 7). Any deployment that cannot dry the beverage residues for free must secure that heat from waste sources or from the plant’s own volatile surplus quantified in Section 3.7 [89].

Eighth, each food residue is represented by one composite sample pooled from several households over a year. Seasonal and between-household variability is averaged rather than quantified, and household residues may differ from the café, catering and shelling-plant streams assumed in the supply chain; the propagated uncertainty is a measurement uncertainty, not a representativeness uncertainty.

Ninth, mass yields and calorific values come from laboratory-scale treatment of milled samples for 30 min under inert gas. Continuous industrial reactors differ in heat transfer, particle size and residence time, and their yields at a given set-point may differ.

Tenth, lignite is displaced on an equal fuel-energy basis. Differences in boiler efficiency between coal and torrefied fuel are examined only as a sensitivity case (Section 3.6).

Eleventh, the volatile stream released during torrefaction is assumed to be combusted on site to supply process heat, as described in Section 3.7. Its emissions of CO, CH_4_ and condensable organics, and the energy actually recoverable from it, were not measured.

## 4. Conclusions

This study quantified the cradle-to-grave carbon footprint of five food-processing residues, spent coffee grounds (SCGs), spent tea leaves (TEAs) and walnut (WAL), pistachio (PIS) and peanut (PEA) shells, and of one forestry residue, spruce wood chips (SPRs), torrefied at 250–550 °C and burned in place of Czech lignite. The base case is a fuel-first allocation in which the agricultural burden is charged to the fuel and the food commodity is credited by a restricted system expansion; cut-off, mass and economic allocation were compared. Fuel properties and received moisture contents were measured directly, and their replicate variances propagated to every reported quantity.

RQ1. All six materials deliver a net saving at their selected treatment temperature: SPR (−1214 ± 23 kg CO_2_-eq t^−1^ of residue), TEA (−1018 ± 23), WAL (−889 ± 19), PIS (−887 ± 22), PEA (−697 ± 21) and SCG (−557 ± 22), avoiding 35.7 to 76.5% of the emissions of the lignite they replace. The ranking follows the supply chain rather than the fuel: once the food credit has offset the farming burden, the supply chain accounts for 46–68% of the remaining burden of TEA and the three nutshells, roasting and brewing for 61% of that of SCG, and drying for 61% of that of SPR.

RQ2. The net balance improves monotonically as the treatment temperature falls, so the carbon optimum always lies at the mildest treatment examined; the fuel-quality constraint that sets the selected temperatures costs up to 391 kg CO_2_-eq t^−1^ of savings and should be declared as such rather than presented as a joint optimum. RQ3. The parameters that decide the outcome are not fuel properties. The waste ratio and the received moisture of a single material, SCG, span the range from a saving of 557 kg CO_2_-eq t^−1^ to an emission of 1450 without one laboratory value changing, whereas the propagated measurement uncertainty of the whole characterisation is 19 to 23 kg CO_2_-eq t^−1^. Collected wet and dried with natural gas, SCG and TEA become net emitters. Resolving drying to the actual water load also shows that the autothermal margin of a plant on woody feedstock is set at the forest landing rather than in the reactor.

RQ4. Only SPR is carbon-negative under every allocation convention tested. For the five food residues, the saving holds under cut-off, economic allocation and system expansion, but under mass allocation, four of the five become net emitters, and without any food credit, the same four do by 1311 to 14,411 kg CO_2_-eq t^−1^, while PEA breaks even. Under system expansion, the farming burden and the food credit cancel out by construction, so the base case is equivalent to charging the residue with the primary-use processing and the primary-product logistics. Reporting that boundary explicitly, rather than selecting the convention that flatters the result, seems to us to be the minimum standard for claims of carbon negativity in waste-to-fuel assessments.

Carbon performance is not by itself a recommendation. TEA ranks second on carbon yet carries above 5% ash at its selected temperature, rising with severity, and should not be proposed for pulverised-fuel co-firing without ash-fusion testing. SCG saves the least and achieves any saving at all only if it reaches the plant already dried. At 65 EUR per tonne of CO_2_, the avoided emissions are worth 36–79 EUR per tonne of residue, while the torrefied fuels cost 7–25 EUR MWh^−1^ against 52.12 EUR MWh^−1^ for brown coal including its allowances, a fuel-cost advantage of 116–200 EUR per tonne. Because the benchmark already carries the allowance cost, the two measures overlap; it is the fuel-cost advantage that must cover the capital and operating cost of the conversion plant. Future work should determine the ash-fusion behaviour of the torrefied products, measure the waste ratio and the as-received moisture of specific residue streams, replace the default lignite factor with a measured composition of the fuel displaced, and extend the assessment to the remaining impact categories. A further case, left for future research, is shelling the nuts in the country of origin and torrefying the shells immediately for local use or for shipment to Europe as a denser fuel, which would remove the shells from the intercontinental freight charged here.

## Figures and Tables

**Table 1:** Feedstocks, waste ratios and agricultural carbon footprints. All masses are on a dry basis. The farming footprint per kilogram of waste follows from Equation (1).

Material	Code	Primary Product	R (kg kg^−1^)	CF Farming (kg CO_2_-eq kg^−1^ Primary)	CF Farming (kg CO_2_-eq kg^−1^ Waste)	Primary Use (kg CO_2_-eq kg^−1^ Waste)	Source
Spent coffee grounds	SCG	Green coffee bean	0.65	2.18	3.354	0.615	[37]; R: [1]
Spent tea leaves	TEA	Made tea	0.70	10.80	15.43	0.214	[38]
Walnut shells	WAL	Walnut, in-shell	0.50	1.10	2.20	0.16	Estimate
Pistachio shells	PIS	Pistachio, in-shell	0.50	2.20	4.40	0.20	[39]; R: [2]
Peanut shells	PEA	Peanut, in-shell	0.35	0.24	0.686	0.171	[40]
Spruce wood chips	SPR	Wood chips	1.00	0.03	0.03	0.02	[41]

R = waste-to-primary mass ratio, on a dry basis; for SCG and TEA, it follows from what roasting and brewing remove (Section 2.1). Primary use comprises roasting and brewing (SCG), tea processing (TEA), cracking and sorting (nuts) and chipping (SPR); packaging of the food product is not charged to the residue (Section 2.3). Farming footprints: SCG, median of a meta-analysis of primary coffee production [37]; TEA, processed tea, which includes part of the processing energy and so partly overlaps the primary-use term [38]; PIS, mean of conventional and low-input orchards, derived from per-hectare data [39]; PEA, farm gate without credit for hull burning [40]; WAL, author estimate, no per-kg value having been found in the literature (it does not affect the base case); SPR, harvesting and chipping [41]. SPR is a dedicated energy feedstock with no food co-product, so R = 1 and no system credit is applied. In the base case, the farming footprint cancels against Credit B (Section 2.4).

**Table 2:** Process parameters and emission factors of the foreground system.

Parameter	Symbol	Unit	Value	Source
Road freight emission factor	EF road	kg CO_2_-eq t^−1^ km^−1^	0.062	Ecoinvent 3.10 [36]
Sea freight emission factor (container > 8000 TEU)	EF sea	kg CO_2_-eq t^−1^ km^−1^	0.0127	DESNZ 2023 [45]
Rail freight emission factor (EU electric)	EF rail	kg CO_2_-eq t^−1^ km^−1^	0.0108	GLEC v3.2 [46]
Distance torrefaction → end user	d3	km	100	Assumption
Czech grid carbon intensity	EF elec	kg CO_2_-eq kWh^−1^	0.38	MPO [47]
Natural-gas emission factor	EF gas	kg CO_2_-eq MJ^−1^	0.202	Ecoinvent 3.10 [36]
Specific drying heat	h dry	MJ kg^−1^ water	3.0	Yun et al. [48]
Target moisture after drying	m t	kg kg^−1^, wet basis	0.05	Assumption
Grinding energy	E grind	MJ kg^−1^	0.12	Literature, 0.08–0.15
Torrefaction energy	E torr	MJ kg^−1^	0.25	Literature, 0.20–0.30
Waste collection	EF collect	kg CO_2_-eq kg^−1^	0.015	Estimate
Lignite emission factor (CO_2_, NCV basis)	EF coal	kg CO_2_ MJ^−1^	0.101	IPCC 2006, Vol. 2, Table 2.2 [44]
CH_4_ + N_2_O from lignite combustion	EF coal,nonCO2	kg CO_2_-eq MJ^−1^	0.00044	IPCC 2006 [44]; AR6 GWP_100_ [35]
CH_4_ + N_2_O from biomass combustion	EF bio,nonCO2	kg CO_2_-eq MJ^−1^	0.0019	IPCC 2006 [44]; AR6 GWP_100_ [35]
Ash transport and landfill	EF ash	kg CO_2_-eq kg^−1^ ash	0.01	Estimate
Functional unit	FU	t dry waste	1	—

Drying heat is supplied by natural gas; grinding and torrefaction are electric. Transport distances and routes are material-specific and are given in Table 3; feedstock moistures and drying energies are in Table 4.

**Table 3:** Origin-specific logistics of Stage 3. The crops are grown in the countries listed; the nuts are shipped in shell and shelled in Europe, SCG and TEA arise in Europe after brewing, and all conversion and combustion take place in the Czech Republic. Distances are typical port-to-port sea routes plus the inland legs; the emission is charged on 1/R tonnes of primary product per tonne of dry residue.

Material	Assumed Origin and Route	Sea (km)	Road at Origin (km)	Rail Port → CZ (km)	1/R (t Primary t^−1^ Residue)	Stage 3 (kg CO_2_-eq t^−1^ Residue)
SCG	Brazil 50%/Vietnam 30%/Colombia 20%; port Hamburg	12,000	300	800	1.54	276
TEA	India, Sri Lanka, China, Kenya; port Hamburg	13,000	300	800	1.43	275
WAL	Mostly EU and neighbours (FR, RO, MD, UA)—road only	—	1500	—	2.00	186
PIS	Iran (Bandar Abbas), also California and Turkey	11,000	300	800	2.00	334
PEA	Argentina/Brazil, also China	12,000	300	800	2.86	513
SPR	Domestic Czech bark-beetle calamity wood	—	100	—	1.00	6

WAL is modelled as road-only within Europe and its eastern neighbourhood, SPR as domestic haulage. Multi-leg accounting follows [49].

**Table 4:** As-received moisture, evaporated water load and drying energy, per kilogram of dry solids, from Equation (6).

Material	Assumed Supply Chain	Moisture as Received (%, Wet Basis)	Water Evaporated (kg kg^−1^ dry)	E Dry (MJ kg^−1^ dry)	Drying (kg CO_2_-eq t^−1^)	Stage 6 Total (kg CO_2_-eq t^−1^)
SCG	brewed spent grounds from cafés, sun-dried before collection	8.3	0.037	0.112	22.7	35.4
TEA	brewed spent leaves from cafés, sun-dried before collection	7.0	0.022	0.067	13.5	26.2
WAL	sun-dried shells from shelling plant	8.0	0.034	0.102	20.6	33.2
PIS	sun-dried shells from shelling plant	6.6	0.019	0.056	11.2	23.9
PEA	sun-dried husks from shelling plant	7.3	0.027	0.080	16.2	28.8
SPR	calamity roundwood seasoned at the landing, then chipped	30.0	0.376	1.128	227.8	240.5

The as-received moisture of the five food residues is the value measured on the characterised sample, which was sun-dried before analysis; spruce is entered at an assumed 30% because its sample was dried beyond the state of field chips. Target moisture after drying is 5% on a wet basis in all cases. The Stage 6 total adds grinding at 0.12 MJ kg^−1^ of electricity, which is 12.7 kg CO_2_-eq t^−1^.

**Table 5:** Fuel properties at the treatment temperature selected for each material, with the standard deviation propagated from the replicate variances by Equation (9). Net calorific values are for the dry state.

Material	T (°C)	NCV Raw (MJ kg^−1^)	NCV Torr (MJ kg^−1^)	Mass Yield (%)	Energy Yield (%)	Energy Residual (MJ kg^−1^)	H/C (molar)	O/C (molar)	Ash (%)
SCG	300	19.95	29.39 ± 0.31	52.3 ± 0.5	77.0 ± 1.4	15.36 ± 0.22	1.159 ± 0.016	0.154 ± 0.008	2.96 ± 0.03
TEA	250	17.11	20.27 ± 0.22	78.3 ± 0.8	92.7 ± 1.7	15.87 ± 0.23	1.171 ± 0.017	0.415 ± 0.009	5.31 ± 0.05
WAL	300	17.13	25.34 ± 0.26	52.0 ± 0.5	77.0 ± 1.4	13.19 ± 0.19	0.932 ± 0.013	0.250 ± 0.008	4.54 ± 0.05
PIS	250	16.59	20.52 ± 0.22	73.0 ± 0.7	90.3 ± 1.6	14.98 ± 0.22	1.273 ± 0.018	0.508 ± 0.009	1.46 ± 0.01
PEA	300	17.25	22.34 ± 0.23	65.5 ± 0.7	84.8 ± 1.5	14.63 ± 0.21	1.061 ± 0.015	0.295 ± 0.008	2.99 ± 0.03
SPR	300	18.27	21.26 ± 0.22	73.6 ± 0.7	85.6 ± 1.5	15.64 ± 0.23	1.193 ± 0.017	0.418 ± 0.009	0.71 ± 0.01

Energy residual is defined by Equation (7). None of the selected products meets the European Biochar Certificate thresholds of H/C < 0.7 and O/C < 0.4 [54]; the full temperature series is shown in Figure 2.

**Table 6:** Nine-stage cradle-to-grave carbon footprint inventory at the selected treatment temperature (kg CO_2_-eq per tonne of dry residue). Positive values are emissions; negative values are credits.

Stage	SCG/300 °C	TEA/250 °C	WAL/300 °C	PIS/250 °C	PEA/300 °C	SPR/300 °C
1. Plantation and farming	3969	15,643	2360	4600	857	50
2. Harvest and waste separation	0	0	0	0	0	0
3. Transport origin → processor	276.4	274.8	186.0	333.9	513.3	6.2
4. Primary use	0	0	0	0	0	0
5. Waste collection	15.0	15.0	15.0	15.0	15.0	15.0
6. Drying and grinding	35.4	26.2	33.2	23.9	28.8	240.5
7. Torrefaction	26.4	26.4	26.4	26.4	26.4	26.4
8. Transport product → user	3.24	4.85	3.23	4.53	4.06	4.56
9. Combustion (CH_4_, N_2_O, ash)	29.4	30.6	25.3	28.6	28.0	29.8
**Gross emissions**	**4355**	**16** **,** **021**	**2649**	**5032**	**1473**	**372**
Credit A—lignite displaced	−1558	−1610	−1338	−1520	−1484	−1587
Credit B—food co-product	−3354	−15,429	−2200	−4400	−686	0
**Net carbon footprint**	**−557**	**−1018**	**−889**	**−887**	**−697**	**−1214**

Stage 2 is folded into Stage 1; the emissions of Stage 4 are contained in the primary-use term of Table 1 and are set to zero here to avoid double counting. Stage 9 charges the CH_4_ and N_2_O emissions from combustion (25–30 kg CO_2_-eq t^−1^) and ash handling (below 0.5 kg CO_2_-eq t^−1^); biogenic CO_2_ is not charged (Section 2.5).

**Table 7:** Net carbon balance and comparison with the lignite reference at the selected treatment temperature; SCG and TEA are also shown collected wet, without solar pre-drying.

Metric	Unit	SCG	TEA	WAL	PIS	PEA	SPR
Fuel energy delivered (NCV)	MJ t^−1^ residue	15,358	15,872	13,189	14,982	14,631	15,640
Residual process burden	kg CO_2_-eq t^−1^	1001	592	449	632	787	372
Credit A—lignite displaced	kg CO_2_-eq t^−1^	−1558	−1610	−1338	−1520	−1484	−1587
Credit B—food co-product	kg CO_2_-eq t^−1^	−3354	−15,429	−2200	−4400	−686	0
Net carbon footprint	kg CO_2_-eq t^−1^	−557 ± 22	−1018 ± 23	−889 ± 19	−887 ± 22	−697 ± 21	−1214 ± 23
Carbon intensity of the delivered fuel	g CO_2_-eq MJ^−1^	65.2	37.3	34.1	42.2	53.8	23.8
Lignite reference (CO_2_-eq)	g CO_2_-eq MJ^−1^	101.4	101.4	101.4	101.4	101.4	101.4
Emissions of displaced lignite avoided	%	35.7	63.2	66.4	58.4	47.0	76.5
Net carbon footprint, collected wet ^a^	kg CO_2_-eq t^−1^	+514	+755	—	—	—	—
Emissions of displaced lignite avoided, collected wet ^a^	%	−33.0	−46.9	—	—	—	—

^a^ SCG at 65% and TEA at 75% as-received moisture, i.e., collected wet. The ‘avoided’ rows are the reduction in carbon intensity relative to the lignite reference; a negative value means the delivered fuel is more carbon-intensive than lignite. In the wet case, water is evaporated in the natural-gas dryer of Stage 6, Equation (6). The uncertainty of the net footprint is one standard deviation propagated from the replicate variances and does not include the uncertainty of the lignite emission factor, which is treated in Section 3.6.

**Table 8:** Net carbon footprint under six accounting conventions (kg CO_2_-eq per tonne of dry residue).

Material	A—Cut-Off	B1—Symmetric Credit	B2—Base Case	C—No Food Credit	D—Mass Allocation	E—Economic Allocation	Spread B1 → B2
SCG	−1449	−833	−557	+2797	+1837	−1431	276
TEA	−1507	−1293	−1018	+14,411	+9635	−1389	275
WAL	−1235	−1075	−889	+1311	+38	−1202	186
PIS	−1421	−1221	−887	+3513	+1046	−1391	334
PEA	−1382	−1210	−697	−11	−902	−1361	513
SPR	−1270	−1214	−1214	−1214	−1214	−1214	0

A = cut-off, the residue entering the system burden-free; B1 = fuel-first with a fully symmetric system expansion that also credits the avoided logistics of the food co-product; B2 = fuel-first with the credit restricted to avoided farming, the base case of this study; C = fuel-first without any co-product credit; D = mass allocation of Stages 1 and 3 between residue and food by dry mass; E = economic allocation by market value. SPR has no food co-product, so B1 = B2 = C = D = E for it.

**Table 9:** Economic assessment: value of the avoided emissions under the EU ETS and delivered fuel cost at the selected treatment temperature.

Item	Unit	SCG	TEA	WAL	PIS	PEA	SPR
ETS value at 50 EUR tCO_2_^−1^	EUR t^−1^ residue	28	51	44	44	35	61
ETS value at 65 EUR tCO_2_^−1^	EUR t^−1^ residue	36	66	58	58	45	79
ETS value at 84 EUR tCO_2_^−1^	EUR t^−1^ residue	47	86	75	75	59	102
ETS value at 150 EUR tCO_2_^−1^	EUR t^−1^ residue	84	153	133	133	105	182
ETS value at 250 EUR tCO_2_^−1^	EUR t^−1^ residue	139	254	222	222	174	304
Feedstock price	EUR t^−1^ raw	30	30	50	50	50	110
Delivered fuel cost	EUR MWh^−1^	7.03	6.80	13.65	12.01	12.30	25.32
Brown coal including allowances	EUR MWh^−1^	52.12	52.12	52.12	52.12	52.12	52.12
Cost headroom	EUR MWh^−1^	45.09	45.32	38.48	40.11	39.82	26.80
Cost headroom	EUR t^−1^ residue	192	200	141	167	162	116

Brown-coal benchmark: 110 EUR t^−1^ at 13.9 MJ kg^−1^ (28.49 EUR MWh^−1^) [19,57] plus an allowance cost of 65 EUR tCO_2_^−1^, the 2024 average [56], at 0.364 t CO_2_ MWh^−1^ (23.63 EUR MWh^−1^); that intensity is the same lignite factor of 0.101 kg CO_2_ MJ^−1^ used for Credit A. ETS rows: 50 EUR is close to the 2024 low, 65 EUR the 2024 average and 84 EUR the 2023 average; 150 and 250 EUR are illustrative. The cost headroom is the difference against the coal benchmark; because that benchmark already includes the allowance cost of the coal, the headroom and the ETS value overlap rather than add (Section 3.8). The headroom must still cover the capital and operating cost of the conversion plant, which is not estimated here.

## Data Availability

The original contributions presented in this study are included in the article/Supplementary Materials. Further inquiries can be directed to the corresponding author.

## Supplementary Materials

The following supporting information can be downloaded at https://www.mdpi.com/article/10.3390/ma19214725/s1. Workbook S1: Complete carbon-footprint inventory of the six torrefied residues (SCG, TEA, WAL, PIS, PEA, SPR), with the measured fuel properties and their replicate variances, the inventory parameters with sources, every formula of the nine-stage model, the full first-order uncertainty propagation, the allocation scenarios A–E, the one-at-a-time sensitivity analysis, the economic assessment and a Python reference implementation that reproduces the headline results.

## Abbreviations

The following abbreviations are used in this manuscript:

SCGs, spent coffee grounds; TEAs, spent tea leaves; WALs, walnut shells; PISs, pistachio shells; PEA, peanut shells; SPRs, spruce wood chips; CF, carbon footprint; EBC, European Biochar Certificate; EU ETS, European Union Emissions Trading System; FU, functional unit; GCV, gross calorific value; GWP, global warming potential; NCV, net calorific value; R, waste-to-primary mass ratio; E_r_, energy residual; TEU, twenty-foot equivalent unit.
